# Anti-Obesity and Anti-Dyslipidemic Effects of *Salicornia arabica* Decocted Extract in Tunisian *Psammomys obesus* Fed a High-Calorie Diet

**DOI:** 10.3390/foods12061185

**Published:** 2023-03-11

**Authors:** Souhaieb Chrigui, Sameh Hadj Taieb, Hedya Jemai, Sihem Mbarek, Maha Benlarbi, Monssef Feki, Zohra Haouas, Ayachi Zemmel, Rafika Ben Chaouacha-Chekir, Nourhène Boudhrioua

**Affiliations:** 1 Laboratoire de Physiopathologie, Alimentation et Biomolécules, LR17ES03, Institut Supérieur de Biotechnologie de Sidi Thabet, Université de la Manouba, Ariana 2020, Tunisia; 2 Laboratory of Clinical Biochemistry, Rabta Hospital, LR99ES11, Faculty of Medicine of Tunis, University of Tunis El Manar, La Rabta, Tunis 1007, Tunisia; 3 Institute for Biomedical and Bioscience Research, the Clore Laboratory, Faculty of Medicine and Health Sciences, University of Buckingham, Hunter Street, Buckingham MK18 1EG, UK; 4 Laboratory of Histology and Cytogenetic LR18ES40, Faculty of Medicine of Monastir, University of Monastir, Av. Avicenne, Monastir 5000, Tunisia; 5 Herbes de Tunisie, El Mansoura Kesra, Siliana 6131, Tunisia

**Keywords:** antioxidant, *Salicornia arabica* decocted extract, high-calorie diet, dyslipidemia, obesity, *Psammomys obesus*

## Abstract

*Salicornia* is a halophyte plant that has been used in traditional medicine for the treatment of scurvy, goiter, and hypertension. It is commercialized in Europe and Asia as fresh salads, pickled vegetables, green salt, or tea powder. This work is the first to assess the potential anti-obesity and anti-dyslipidemic effects of *Salicornia arabica* decocted extract (SADE). SADE was characterized by its significant in vitro radical scavenging activity (using DPPH and ABTS assays). The effect of SADE on food intake, weight loss, serum biochemical parameters, liver and kidney weights, adiposity index and on liver histology was investigated in the Tunisian gerbil *Psammomys obesus* (*P. obesus*), which is recognized as a relevant animal model of human obesity and diabetes. *P. obesus* animals were firstly randomly divided into two groups: the first received a natural low-calorie chow diet (LCD), and the second group received a high-calorie diet (HCD) over 12 weeks. On day 90, animals were divided into four groups receiving or not receiving SADE (LCD, LCD + SADE, HCD, and HCD + SADE). If compared to the HCD group, SADE oral administration (300 mg/kg per day during 4 weeks) in HCD + SADE group showed on day 120 a significant decrease in body weight (−34%), blood glucose (−47.85%), serum levels of total cholesterol (−54.92%), LDL cholesterol (−60%), triglycerides (−48.03%), and of the levels of hepatic enzymes: ASAT (−66.28%) and ALAT (−31.87%). Oral administration of SADE restored the relative liver weight and adiposity index and significantly limited HCD-induced hepatic injury in *P. obesus*. SADE seems to have promising in vivo anti-obesity and anti-dyslipidemic effects.

## 1. Introduction

Obesity and overweight result from an imbalance between consumed calories, basal metabolism, and energy expenditure [1]. They are characterized by excessive accumulation of fat in adipose tissue, liver, pancreatic islets, muscles and other metabolism-involved organs, which can be harmful to health [2]. The modern lifestyle involving a high-calorie diet (HCD) and less physical activity contributes to the concomitant development of obesity and dyslipidemia. Dyslipidemia results from lipid metabolic changes and is characterized by elevated concentrations of total cholesterol (TC), low-density lipoprotein cholesterol (LDL), and triglycerides (TG), and low levels of high-density lipoprotein cholesterol (HDL) [3]. Obesity, dyslipidemia and their complications such as diabetes mellitus, hypertension and cardiovascular diseases are one of the major causes of comorbidity and the excess of mortality [3,4,5,6,7]. The treatment of obesity has proven hugely resistant to therapy, with anti-obesity medications which often showed insufficient efficacy, dubious safety and gastrointestinal side effects (nausea, diarrhea, vomiting, and constipation) [8]. Natural anti-obesity products (biomolecules, plant crude extracts, mixture of fruit or plant crude extracts) were reported to be an excellent alternative strategy for developing effective and safe anti-obesity and anti-dyslipidemic agents [1,9,10,11,12,13,14,15]. For example, it was shown that long-term *Huangshan Maofeng* green extract supplementation remarkably reduced excessive fat accumulation, increased gut microbiota diversity, restored the relative abundance of the microbiota responsible for producing short-chain fatty acids and reduced hyperlipidemia and hepatic steatosis in rats [16]. Antioxidants and particularly phenolic compounds (simple phenolic acids, curcumins, flavonoids, anthocyanin, and catechins) were reported to have potent anti-obesity and dyslipidemic effects. Anti-obesity and anti-dyslipidemic effects of phenols were essentially related to the enhancement of antioxidant defense, the inhibition of cholesterol absorption and lipogenic and adipogenic activities [17]. Halophytes, native plants of saline ecosystems, present economic and ecological interests because of their high salt tolerance and are an important source of bioactive compounds, particularly phenols [18]. They are evaluated as a promoting candidate for culinary and pharmaceutical applications [19]. The genus *Salicornia* is a halophyte belonging to the *Chenopodiaceae* family, including about thirty species of succulent annual hygro-halophyte plants. *Salicornia* genus has recently been commercialized in Europe and Asia as a staple food, and it is used in green salads for its saltiness [20] or as an ingredient in various recipes as tea powder, seasoned vegetable, makgeolli, pickled vegetables, vinegar and fermented food [21]. Various promoting therapeutic applications have been reported for *Salicornia* species: *Salicornia herbacea* and *Salicornia bigelovii* are in particular used against oxidative stress, inflammation gastroenteritis, cancer, diabetes, asthma and hepatitis [21] and in the treatment of various diseases such as obesity, hyperglycemia and hyperlipidemia [22,23]. *Salicornia herbacea* supplementation reduces fat accumulation in the liver and regulates hepatic triglycerides [24], decreases lead-induced oxidative stress, and exerts cytoprotective action [25]. *Salicornia arabica* (*S. arabica*) lipid extract was reported to induce a protective effect against cadmium-induced erythrocyte damage. In vitro, the antioxidant properties of *S. arabica* polysaccharides and lipid extracts were also examined [26,27], whereas the potential in vivo protective effect of antioxidants in the decocted extract of *S. arabica* is not yet explored. *Psammomys obesus* (*P. obesus*), *Muridae*, *Gerbillidae* is a desert gerbil of particular interest because, in its native habitat, *P. obesus* feeds on low-caloric vegetation remains healthy when in captivity and is subjected to nutritional stress induced by a high-calorie laboratory diet, resulting in the development of obesity, dyslipidemia, diabetes [28], and diabetic retinopathy [29]. *P. obesus* is known as a reference animal model of nutritionally-induced obesity and its complications [28].

The aim of this work is to examine the anti-obesity and anti-dyslipidemic effects of the antioxidant decocted extract of *S. arabica* in *P. obesus* fed a high-fat calorie diet. To the best of our knowledge, this is the first work reporting in vivo anti-obesity and anti-dyslipidemic effects of supplemented *S. arabica* decocted extract (SADE) in HCD induced -obesity and dyslipidemia in rats. The effects of SADE on body weight, food intake, energy intake, serum biochemical parameters, liver and kidney relative weights, adiposity index and on the histology of the liver tissue were assessed.

## 2. Materials and Methods

### 2.1. Plant Material

*Chenopodiaceae* plant material (*S. arabica*) was collected and identified at the Biology Agronomy and Plant Biotechnologies Department at the National Agronomic Institute, University of Carthage, Tunisia.

### 2.2. High-Calorie and Low-Calorie Diets

A high-calorie standard laboratory chow (EL BADR, Bizerte, Tunisia) supplemented with 30% sugar, 30%fat (corn oil) and saline water (NaC1 0.9%) was used as a high-calorie diet (HCD). The aerial part of *Chenopodiaceae* plant material (*S. Arabica*) was used as a natural low-calorie diet (LCD).

### 2.3. Proximate Chemical Composition of Low and High-Calorie Diets

The Association of Official Analytical Chemists (AOAC) method [30] was used to determine the nutritional compositions and energetic values of LCD and HCD. Samples were weighed and dried at 105 °C for 24 h for moisture content determination. Ash content was measured by using a muffle furnace (Nabertherm GmbH, Lilienthal, Bremen, Germany) at 550 °C for 5 h. Total protein content was determined by the Kjeldahl method and calculated by multiplying the nitrogen content by 6.25. Fat content was determined by using the Soxhlet method, with hexane as a solvent. Carbohydrate content was estimated by the difference of mean experimental values, i.e., 100 − (sum of percentages of moisture, ash, protein and fat) [31]. The sample weight was measured by an analytical balance (Ohaus Corporation, Parsippany, NJ, USA) having a precision of ±10^−4^ g. Energetic values (EV) of LCD and HCD were estimated on the basis of protein, fat and carbohydrates content as follows [32]:

(1) $$EV\;\left(\frac{kcal}{g}\right)=4\times Protein\;content+4\times Carbohydrate\;content+9\times Fat\;content$$

### 2.4. Preparation of Decocted Salicornia arabica Extract

The aerial part of *S. arabica* was dried in the dark in the oven at 40 °C. After drying, the plant material was crushed in a mixer mill (Isolab, Laborgeraete GmbH, Germany), then 100 g of *S. arabica* powder was added to 1000 mL of distilled water and boiled for 15 min. It was then left to cool at room temperature for 20 min. The extract was centrifuged for 10 min at 5000 rpm, and the supernatant was filtered through Whatman filter paper. The resulting decoction was concentrated using a rotary evaporator, frozen and then freeze-dried (Biobase, BK-FD12P, Jinan, China). The obtained freeze-dried decocted extract (SADE) was vacuum packed and stored at −30 °C until use.

### 2.5. In Vitro Antioxidant Properties of Decocted Salicornia arabica Extract

The total phenols content (TPC), total flavonoids content (TFC), and the radical scavenging activities of SADE were determined.

#### 2.5.1. Determination of Total Phenols Content

The determination of total phenols content (TPC) was carried out according to the Folin–Ciocalteu method modified according to M’hiri et al. [33]. The sample was added to Folin–Ciocalteu reagent and Na_2_CO_3_ solution and placed in a water bath at 40 °C for 30 min before spectrophotometric analysis (PEAKII UV, C7200S, USA). Total phenolic content was determined colorimetrically at 765 nm and expressed as mg of Gallic Acid Equivalent (GAE) per g of freeze-dried SADE.

#### 2.5.2. Determination of Total Flavonoids Content

The determination of total flavonoids content (TFC) was carried out by spectrophotometric method as described by M’hiri et al. [33] with aluminum trichloride. First, 0.5 mL of *S. arabica* decocted extract (SADE) was placed in a 5 mL plastic tube, then 2.5 mL of distilled water, followed by 0.15 mL of NaNO_2_ (5%), was added. After 5 min, 0.15 mL of AlCl_3_ (10%) was added, and finally, 1 mL of NaOH (1M) was added another 5 min afterward. The volume was made up to 5 mL with distilled water. The solution was mixed, and the absorbance was measured at 510 nm using a spectrophotometer (PEAKII UV, C7200S, Houston, TX, USA). Total flavonoid content was expressed in mg of Quercetin Equivalent per g of freeze-dried SADE.

#### 2.5.3. Determination of In Vitro Antioxidant Radical Scavenging Activities

The free radical scavenging activity of SADE extract was determined using a 1.1-diphenyl-2-picrylhydrazyl (DPPH) assay [34] and a 2 2′-azino bis 3-ethylbenzothiazoline-6-sulfonic acid (ABTS) assay with minor modifications as reported by M’hiri et al. [33]. For the DPPH assay, the DPPH radical scavenging activity of the extract was assessed by measuring the absorbance at 515 nm using a spectrophotometer (PEAKII UV, C7200S, Houston, TX, USA). For the ABTS assay, ABTS radical scavenging activity was determined by measuring the absorbance at 734 nm. DPPH and ABTS radicals scavenging activities were expressed as mg Trolox Equivalent, TE eq. per g of freeze-dried SADE.

### 2.6. Animals

The Tunisian *P. obesus* animals used in the trials were captured in Southern Tunisia (Bouhedma region, located at 34°25′0″ N, 9°30′0″ E). The animals were then taken into captivity at the laboratory animal facility and left for a week for acclimation in a large aquarium and furnished space with sand, nearly similar to their natural environment. After acclimation, adult male *Psammomys obesus* (*P. obesus*) aged eight weeks and having an average body weight of 101 ± 8 g. Animals placed in individual cages (40 cm × 25 cm × 17 cm) and maintained during the experiment of 120 days under standard and controlled environmental conditions (temperature 22–25 °C, hygrometry 60–70%) with a light on a photoperiod 12/12 [35].

### 2.7. Experimental Design

During the first week corresponding to the animals’ adaptation to life in captivity, animals were exclusively fed on their natural *Chenopodiaceae* halophilic plant, LCD. After the week of adaptation, the 40 *P. obesus* were first randomly divided into two groups:

- Control group: received the natural vegetable diet of *P. obesus,* which is considered in this work as a natural low–calorie diet (LCD)
- HCD group: received the high-calorie diet, rich in carbohydrates and fat
- On the 90th day, each group of animals (LCD and HCD) was divided into two groups each, as follows:
- LCD: used as a negative control: received the natural low-calorie vegetable diet
- LCD + SADE: used as positive control: received *Chenopodiaceae* with oral administration of a dose of 300 mg SADE/kg per day
- HCD: fed with HCD without administration of SADE
- HCD + SADE: fed with HCD with oral administration of a dose of 300 mg SADE/kg per day.

The four animal groups received food and water *ad-libitum* during the 120-day period.

The choice of SADE dose of 300 mg SADE/kg per day was made on the basis of published data using other halophyte plant extracts for in vivo experimentations [36,37].

The percentage of the *P. obesus* initial body weight, % Pi, was determined according to Equation (2) and was presented bi-monthly. The body mass index (BMI) was determined monthly. It was assessed by dividing the body weight (g) by the square of the nose-anus length (cm) of *P. obesus* [38].

(2) $$\%\;\mathrm{Pi}=\frac{\mathrm{Pt}\times 100}{\mathrm{Pi}}$$

(3) $$\mathrm{BMI}\;(g/cm^{2})=\frac{\mathrm{Body}\;\mathrm{weight}\;\left(g\right)}{\mathrm{Body}\;\mathrm{length}h^{2}\;\left(\mathrm{cm}\right)}$$

where Pt is *P. obesus* weight at time t and Pi is its initial weight.

Animals were considered obese when the average weight gain of the animal at a given time was equal to or superior to 150% [35]. Preliminary investigations showed that this criterion is in agreement with the measurement of *P. obesus’* BMI, which was higher than 0.68 g/cm^2^ for obese animals [38].

The nutritional status was determined by calculating food consumption (FC), energy intake (EI), energy intake, EI and feed efficiency (FE) [39,40,41].

Daily food consumption, or FC (g/day), was calculated as follows:

(4) FC (g/day)= quantity of food supplied− quantity of food remaining after 24 

Average values of daily food intake (FI) and energy intake (EI) were determined for each animal group.

Food intake, FI (%/day), was calculated as follows:

(5) $$\mathrm{FI}\;\left(\%/\mathrm{day}\right)=\frac{\mathrm{FC}\;\left(g/\mathrm{day}\right)}{\mathrm{Animal}\;\mathrm{weight}\;\left(g\right)}\times 100$$

Daily energy intake, EI (kcal), was calculated as follows:

(6) $$\mathrm{EI}\;\left(\mathrm{kcal}/\mathrm{day}\right)=\mathrm{FC}\;\left(g/\mathrm{day}\right)\times \mathrm{dietary}\;\mathrm{metabolizable}\;\mathrm{energy}\left(\frac{\mathrm{kcal}}{g}\right)\;$$

where food consumption (FC) was weighed, and dietary metabolized energy was calculated according to corresponding energetic values.

Feed efficiency (FE) was expressed in % and was defined as the ability of animals to convert feed energy consumed in body weight and was measured by dividing body weight gain (g) by the total energy intake (kcal) and multiplying by 100 [39,40,41]:

(7) $$\mathrm{FE}\;\left(\%\right)=\frac{\mathrm{Mean}\;\mathrm{body}\;\mathrm{weight}\;\mathrm{gain}\;\left(g\right)}{\mathrm{Total}\;\mathrm{energy}\;\mathrm{intake}\;\left(\mathrm{kcal}\right)}\times 100$$

### 2.8. Blood Sampling and Serum Biochemical Parameters Analyses

Blood glucose level was measured bi-monthly. Blood was collected by retro-orbital sinus puncture with a capillary hematocrit and was estimated using an Accu-Check Blood Glucose Meter (Roche, Manheim, Germany). The serum is obtained by centrifugation of the blood at 5000 rpm for 15 min, 4 °C, aliquot, and stored at −30 °C until used for analysis. Serum concentrations of total cholesterol (TC), total triglycerides (TG), low-density lipoprotein (LDL), high-density lipoprotein (HDL), aspartate aminotransferase (ASAT) and alanine aminotransferase (ALAT)were assessed by the enzymatic colorimetric method by an Architect C8000 analyzer (Abbott Laboratories, Abbott Park, IL, USA) using the respective reagent kits [42].

Furthermore, the atherogenic index (AtI) was calculated for different animal groups. It is defined as the ratio of LDL (TC-HDL) and HDL according to the Friedewald equation.

(8) $$\mathrm{AtI}=\frac{\left(\mathrm{TC}-\mathrm{HDL}\right)}{\mathrm{HDL}}$$

*P. obesus* animals were considered dyslipidemic when TC ≥ 2.00 g/L and/or LDL ≥ 1.60 g/L and/or TG ≥ 1.50 g/L and/or HDL < 0.40 g/L.

### 2.9. Animal Sacrifice and Organs Sampling

After four months of experimentation, the animals were weighted and sacrificed by decapitation. Immediately after sacrifice, the liver, the kidneys and the adipose tissue were excised and washed with 0.9% NaCl, and the relative weights were determined.

Adiposity index (I_a_) was calculated as follows [39]:

(9) $$I_{a}=\frac{\mathrm{AT}\;\left(g\right)}{\mathrm{Animal}\;\mathrm{weight}\;\left(g\right)}\times 100$$

where AT (g) is the weight of adipose tissue.

### 2.10. Animal Welfare and Ethics Statement

The present experimental protocol was approved by the National Ethical Committee on Medical and Animal Research of the National Veterinary Medicine School, E.N.M.V of Tunisia (Approval Number: CEEA-ENMV 23/20). The study was performed in accordance with the “Guide for the Care and Use of Laboratory Animals” published by the US National Institutes of Health (NIH publication No. 85–23, revised 1996). All efforts were made to minimize animal suffering and reduce the number of animals used.

### 2.11. Histological Observation of the Liver

Liver samples were fixed in a 10% buffered neutral formalin solution and were transferred into an automatic processor (Leica TP 1020, Buffalo Grove, IL, USA) where they were dehydrated in a graded ethanol series, cleared in xylene, and embedded in paraffin wax. Samples were sectioned at 5 μm thickness by using a rotary microtome (Medite M380, Burgdorf, Germany). The sections were stained with hematoxylin and eosin (H&E) and then were examined using a Leica light microscope (Leica DM750, Wetzlar, Germany), and provided with a camera (Leica ICC50, Wetzlar, Germany). For each liver specimen, tissue changes were examined in 10 randomly selected areas. The microscopic appearance of the liver tissues was examined for fatty vacuolation, hepatocyte necrosis, hepatocyte ballooning, massive micro and macrovesicular, intracellular lipid droplets in hepatocytes and inflammatory cell infiltration. The number of apoptotic cells, necrotic cells and lipid droplets were measured using the particle sizing function provided by the ImageJ software version 1.53 (Rasband, ImageJ, National Institutes of Health, Bethesda, MD, USA). Counting cells was assessed in triplicate using the following equation:

(10) $$\mathrm{Counting}\;\mathrm{cells}\;\left(\%\right)=\frac{\left(\mathrm{count}\;\mathrm{target}\;\mathrm{cells}\right)}{\left(\mathrm{Total}\;\mathrm{number}\;\mathrm{of}\;\mathrm{hepatic}\;\mathrm{cells}\right)}\times 100$$

### 2.12. Statistical Analysis

Results were expressed as mean values ± standard deviation (SD). Multiple-group analysis using ANOVA, with a post-hoc test, was used to analyze the statistical significance between parameters measured for different animal groups. Differences were considered significant when *p* < 0.05. Principal components analysis (PCA) was performed on measured average body weight gain (% Pi) and biochemical parameters (TC, TG, HDL, LDL, ALAT, ASAT, and AtI) to elucidate significant differences between animal groups (LCD, LCD + SADE, HCD, and HCD + SADE) on day 0, day 90 (the beginning of SADE administration) and day 120 (the end of SADE administration and of the experiment). The number of dimensions considered for the PCA was chosen to equal two in order to allow meaningful interpretations of the results. All statistical analyses were performed using SPSS statistical software version 22.0 (SPSS Inc., Chicago, IL, USA).

## 3. Results and Discussion

### 3.1. Proximate Chemical Composition and Energetic Values of Low and High-Calorie Diets

Table 1 shows the proximate composition of LCD and HCD. The HCD is characterized by EV of 4.50 kcal/g with the following composition: 9.11% moisture, 58.33% carbohydrates, 2.83% ash, 10.82% proteins, and 18.91% fat.

The LCD corresponding to the areal part of *S. arabica* is characterized by high ash content (8.42 ± 0.15 g/100 g wb) and moisture content (81.63 ± 0.69 g/100 g wb) and a low EV (0.42 ± 0.03 kcal/g wb). A similar composition was reported for the *Chenopodiaceae* plant, *Salsola foetida*, with an EV of 0.4 kcal/g [38], *S. bigelovii,* with an EV of 0.3 kcal/g [31], and *S. herbaceae* with an EV of 0.44 kcal/g [43]. The determined EV of HCD is also similar to that used in similar experiments varying from ~3.70 kcal/g [38,44] to ~4.00 kcal/g [41,45].

### 3.2. Phenols, Flavonoid Contents, and In Vitro Radical Scavenging Activities of SADE

Total phenolic, total flavonoid contents (TPC, TFC), DPPH, and ABTS radical scavenging activities of *S. arabica* decocted extract (SADE) are presented in Table 2.

SADE is characterized by a TPC of 20.5 ± 0.3 mg GAE/g and an antioxidant activity of 3.2 ± 0.1 mg Trolox/g of extract (DPPH assay) and 17.30 ± 0.65 mg TE/g (ABTS assay). Spectrophotometric determination methods of total phenols, flavonoids content, and antioxidant activities in plant extracts may be subject to artifacts due to the interaction with other compounds, such as non-phenolic pigments. Nevertheless, these methods allow the screening of plant extracts according to their antioxidant potential in comparison with other plant extracts assessed by the same methods [46]. TPC and TFC in *S. arabica* decocted extract were high compared to some known halophyte plants assessed with spectrophotometric methods. Chikhi et al. [47] reported that the aqueous extract of *Atriplex halimus* has a phenolic content of 12.4 mg GAE/g extract, and Kim et al. [48] reported that the aqueous extract of *Salicornia europaea* contains 11.6 mg QE/g extract. ABTS scavenging capacity of SADE is similar to that of *S. europea* originating from Italy (ABTS = 15.1 mg TE/g) reported by Costa et al. [49]. Halophyte plant extracts are characterized by promising in vitro antioxidant activities, as reported by many authors [19,22,50]. Phenolic acids such as ferulic acid, cinnamic acid, chlorogenic acid and coumaric acid were reported as the main phenolic compounds in many halophytes [18,37,51,52].

### 3.3. Effects of SADE on Energy, Food Intake, Energy Efficiency, Body Weight Change and Body Mass Index

#### 3.3.1. Effects of SADE on Food, Energy Intakes and Energy Efficiency

**Figure 1:**
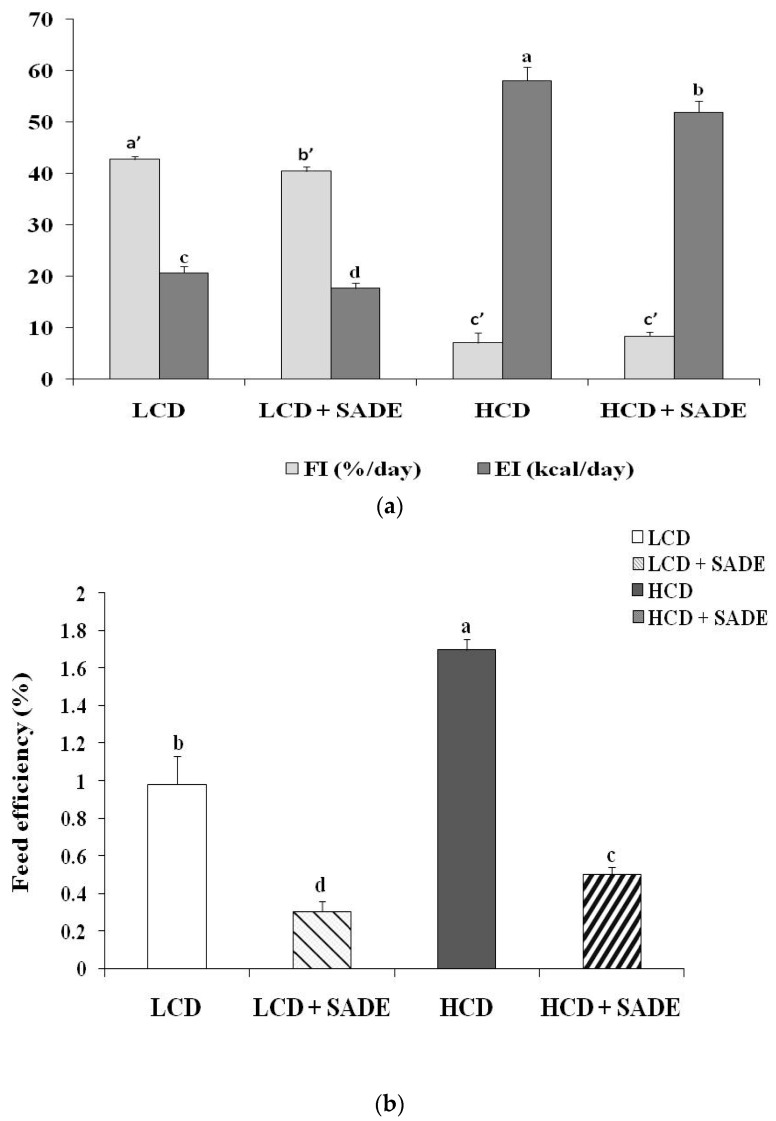
(**a**) Effect of SADE on average food intake (FI) and energy intake (EI) in *P. obesus*. LCD: group fed with natural low-calorie vegetable diet, LCD + SADE: LCD group treated with *S. arabica* decocted, HCD: rats fed with high-calorie diet and HCD + SADE: rats fed with high-calorie diet treated with *S. arabica* decocted extract. Results are presented as means ± SD for ten rats in each group. Means not sharing the same superscript letters (a, b, c, d, a’, b’, c’) are significantly different between groups (Tukey’s post hoc test, *p <* 0.05). “a” denotes the highest value, and “d” represents the lowest value for EI. “a’” denotes the highest value and “c” represents the lowest value for FI. (**b**) Effect of SADE on average feed efficiency, FE in *P. obesus*. LCD: animals group fed with natural low-calorie vegetable diet, LCD + SADE: control treated with *S. arabica* decocted, HCD: rats fed with high-calorie diet and HCD + SADE: rats fed with high-calorie diet treated with *S. arabica* decocted extract. Results are presented as means ± SD for ten rats in each group. Means not sharing the same superscript letters (a, b, c, d) are significantly different between groups (Tukey’s post hoc test, *p <* 0.05). “a” denotes the highest value and “d” represents the lowest value.

The average daily food intakes (FI) of the LCD group and LCD + SADE group were 42.77 ± 0.62%/day and 40.47 ± 0.90%/day, respectively (Figure 1a). The values of FI of HCD and HCD + SADE-fed animals were significantly (*p <* 0.05) lower (7.11 ± 1.46%/day for HCD and 8.41 ± 0.85%/day for HCD + SADE) than those of the LCD and LCD + SADE groups. Due to the high-calorie value of HCD (4.50 kcal/day) compared to a natural low-calorie diet (0.42 kcal/g), the daily EI of both HCD (58.11 ± 2.58 kcal/day) and HCD + SADE-fed animals (51.94 ± 2.11 kcal/day) are higher than EI of LCD group (20.72 ± 1.19 kcal/day) and LCD + SADE-fed animals (17.75 ± 0.48 kcal/day) (Figure 1a).

According to the feed efficiency percentage, FE (%) (Figure 1b), the LCD +SADE group underwent a significant decrease as compared to the LCD group (*p* < 0.05). The HCD group demonstrated a significantly higher FE percentage in comparison to the HCD + SADE (*p* < 0.05) and LCD group (*p* < 0.05). The HCD + SADE exhibited a significant decrease in FE ratio as compared to the LCD group (*p* < 0.05) (Figure 1b). SADE administration seems to decrease food and energy intake in *P. obesus*.

In addition, FE was higher in the HCD group (+73.46%) than in the LCD group. This difference is two folds higher than those reported by Ferron et al. [39] and Rocha et al. [41] for Wistar rats fed HCD. FE value assessed in the HCD group (1.70 ± 0.06%) is lower than that obtained by Novelli et al. [40] for Wistar rats (5.4 ± 0.8%). This difference can be due to different body weight gain in the different used animal models.

#### 3.3.2. Effects of SADE on Body Weight Change

**Figure 2:**
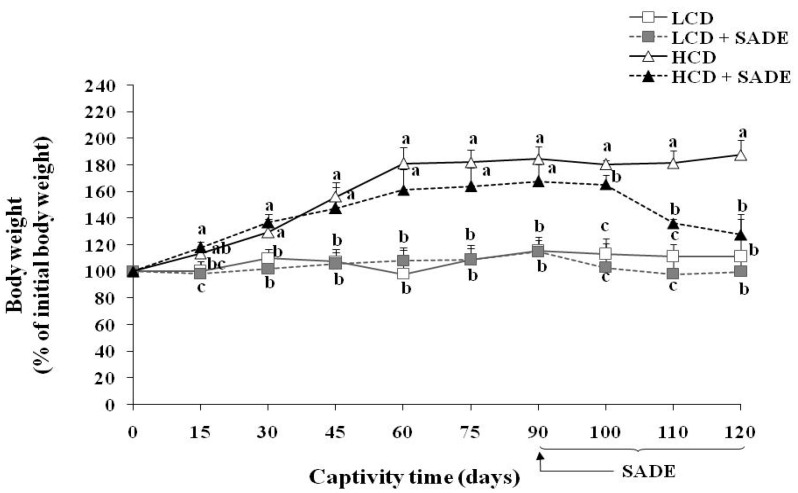
Body weight in % of initial weight in LCD: low-calorie diet, LCD + SADE: animals group treated with *S. arabica* decocted, HCD: rats fed with high-calorie diet and HCD + SADE: rats fed with high-calorie diet treated with *S. arabica* decocted extract. Results are presented as means ± SD for ten rats in each group. Means not sharing the same superscript letters (a, b, c) for each captivity time are significantly different between groups (Tukey’s post hoc test, *p* < 0.05). “a” denotes the highest value and “c” represents the lowest value.

Figure 2 shows the body weight change in percentage (% Pi) for the different groups of *P. obesus* during the experimental period. As expected, animals in HCD fed group showed significantly higher body weight change than those fed with a normal low-calorie diet (LCD). The % Pi of the LCD and LCD +SADE animals remained almost constant during the 120 days of the experiment. However, after 30 days, the % Pi of the HCD and HCD + SADE rats were significantly higher than those of the LCD and LCD + SADE rats (*p* < 0.05). The % Pi of the HCD animals remained significantly higher (187.58 ± 12.47%; *p* < 0.05) than that of the LCD and LCD +SADE animals during 120 days of the experiment. The *P. obesus* fed HCD and administered with SADE on day 90 showed a marked decrease of %Pi (123.81 ± 7.77%) by 34% after 120 days if compared to HCD group (*p* < 0.05) and was not significantly different if compared to the LCD and LCD + SADE groups (respectively, 111.27 ± 11.72% and 98.85 ± 4.52%) (Figure 2). These observations confirm that obesity was successfully induced after the first month of the HCD diet application. HCD + SADE-fed *P. obesus* showed a decrease in animal weight, indicating that SADE was effective in averting weight gain. This is in agreement with the result above, indicating the decrease of EI of the HCD + SADE group compared to the EI of the HCD group (Figure 1a). FE value was significantly lower in the HCD + SADE group (Figure 1b), indicating that SADE prevents animal body weight increase.

Used HCD (4.50 kcal/g) allows a fast establishment of obesity in *P. obesus*. Indeed, generally, more than one month is necessary to establish obesity in such animal models. Several studies showed that the sand rat develops obesity after two to three months of HCD (3.25 kcal/g and 3.85 kcal/g, respectively) [44,53]. HCD administration for up to three months caused significant metabolic changes in *Meriones shawi* rats and resulted in the development of obesity [54]. In addition, some studies using Wistar rats [55] reported that the HCD (3.65 kcal/g) induces an increase in body weight and establishment of obesity from two to six months of HCD. Besides, the results of the present study are in agreement with those of Rahman et al. [36], who reported that the oral administration of 250 and 500 mg/kg of *S. europaea* for 12 weeks resulted in a significant reduction in the body weight of the Sprague–Dawley rats fed on HCD.

#### 3.3.3. Effects of SADE on Body Mass Index

**Figure 3:**
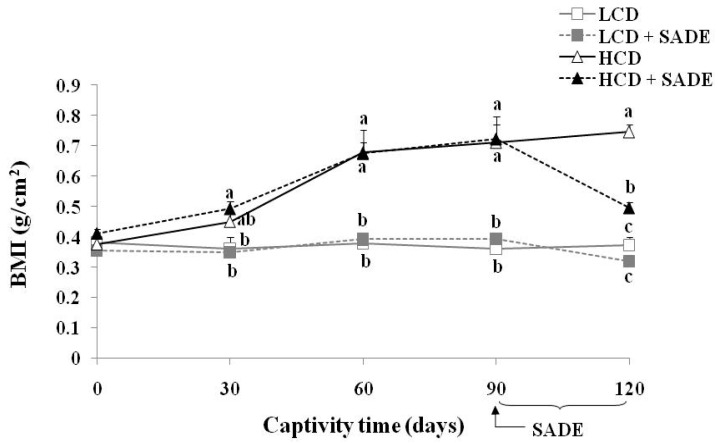
Body mass index, BMI (g/cm^2^) in *P. obesus* in the different animal groups: LCD: animals group fed with natural low-calorie vegetable diet, LCD + SADE: LCD treated with *S. arabica* decocted, HCD: rats fed with high-calorie diet and HCD + SADE: rats fed with high-calorie diet treated with *S. arabica* decocted extract. Results are presented as means ± SD for ten rats in each group. Means not sharing the same superscript letters (a, b, c) for each captivity time are significantly different between groups (Tukey’s post hoc test, *p* < 0.05). “a” denotes the highest value and “c” represents the lowest value.

BMI in rats is an easy and reproducible anthropometric assessment of obesity [56]. When BMI is above 0.68 g/cm^2^, *P. obesus* are considered obese, and there is an increased risk of developing metabolic syndrome such as dyslipidemia by an increase in lipid biomarkers [28]. As shown in Figure 3, there were no significant BMI differences between the different animal groups at the beginning of the experiment.

However, HCD and HCD + SADE groups led to a strong increase in BMI from days 30 to 90 (*p* < 0.05), whereas LCD and LCD + SADE groups were similar and stayed roughly constant until the end of the experiment. After oral administration of SADE, from day 90 to day 120, BMI slightly decreased on day 120 in LCD + SADE group ranging from 0.39 ± 0.02 g/cm^2^ (day 90) to 0.32 ± 0.02 g/cm^2^ (day 120) compared to the LCD group and the difference was statistically significant (*p* < 0.05). A high significant decrease (*p* < 0.05) in BMI on day 120 was noticed in HCD + SADE animals ranging from 0.72±0.07 g/cm^2^ (day 90) to 0.50 ± 0.02 g/cm^2^ (day 120) compared to the HCD group (0.75 ± 0.02 g/cm^2^). Figure 3 shows progressive changes in BMI in all groups of *P. obesus* during the experimental period. None of the animals in the HCD group was obese before the first thirty days, which indicates that BMI stayed normal and has not attained 0.68 g/cm^2^. However, HCD led to a rapid body weight change and accelerated the development of obesity after the first month. A similar value of BMI was reported for Wistar rats and *P. obesus* by Mashmoul et al. [56] (0.76 ± 0.05 g/cm^2^) and Gouaref et al. [38] (0.67 ± 0.03 g/cm^2^).

### 3.4. Effects of SADE on the Relative Weight of Liver and Kidney and Adiposity Index Changes

**Figure 4:**
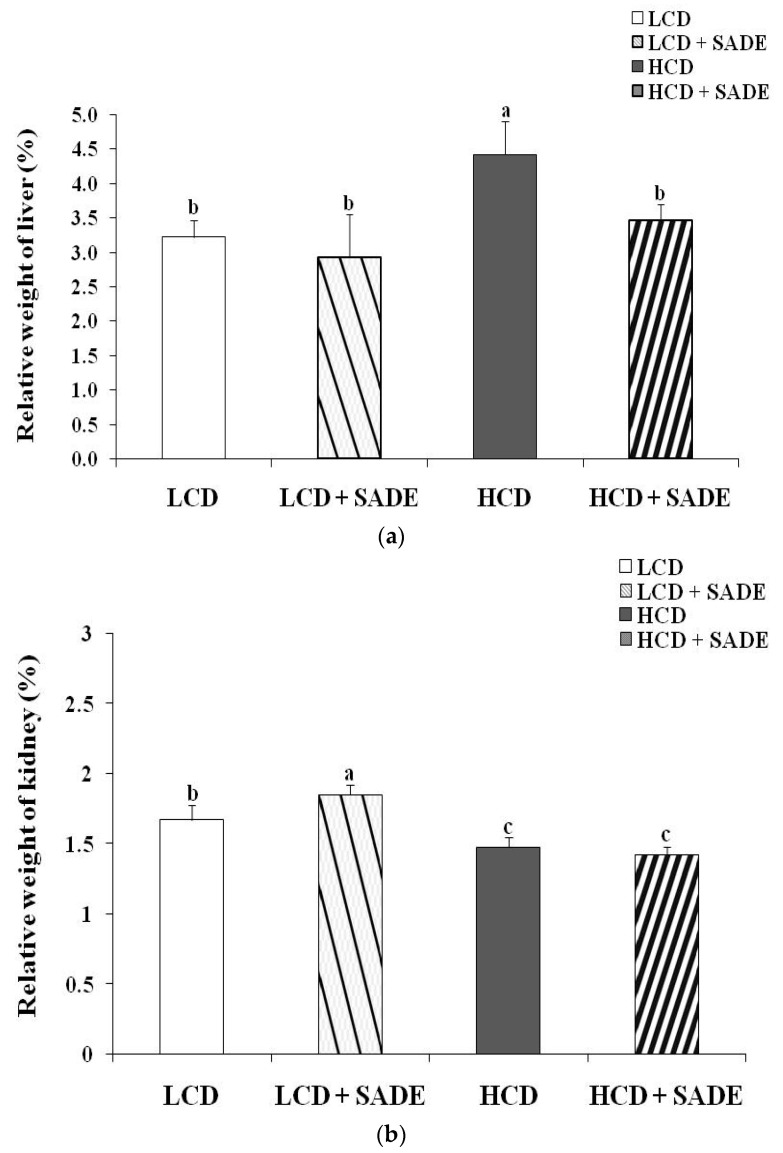

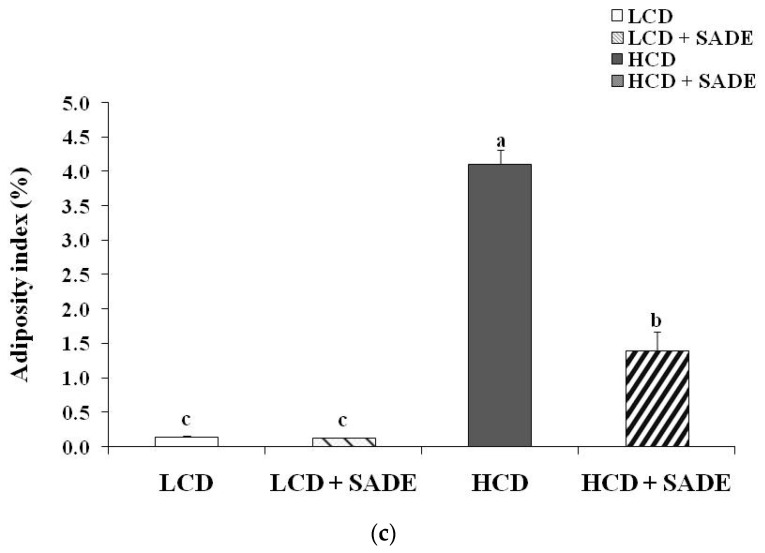
(**a**) Effect of SADE on the relative weight of the liver in *P. obesus* measured on day 120 in the different animal groups. LCD: animal group fed with natural low-calorie vegetable diet, LCD + SADE: control treated with *S. arabica* decocted, HCD: rats fed with high-calorie diet and HCD + SADE: rats fed with high-calorie diet treated with *S. arabica* decocted extract. Results are presented as means ± SD for ten rats in each group. Means not sharing the same superscript letters (a, b) are significantly different between groups (Tukey’s post hoc test, *p* < 0.05). “a” denotes the highest value and “b” represents the lowest value. (**b**) Effect of SADE on the relative weight of the kidney in *P. obesus* measured on day 120 in the different animal groups. LCD: animal group fed with natural low-calorie vegetable diet, LCD + SADE: LCD treated with *S. arabica* decocted, HCD: rats fed with high-calorie diet and HCD + SADE: rats fed with high-calorie diet treated with *S. arabica* decocted extract. Results are presented as means ± SD for ten rats in each group. Means not sharing the same superscript letters (a, b, c) are significantly different between groups (Tukey’s post hoc test, *p <* 0.05). “a” denotes the highest value and “c” represents the lowest value. (**c**) Effect of SADE on the relative weight of adiposity index in *P. obesus* measured on day 120 in the different animal groups. LCD: animal group fed with natural low-calorie vegetable diet, LCD + SADE: LCD group treated with *S. arabica* decocted, HCD: rats fed with high-calorie diet and HCD + SADE: rats fed with high-calorie diet treated with *S. arabica* decocted extract. Results are presented as means ± SD for ten rats in each group. Means not sharing the same superscript letters (a, b, c) are significantly different between groups (Tukey’s post hoc test, *p* < 0.05). “a” denotes the highest value and “c” represents the lowest value.

Figure 4 shows the effect of SADE on the relative liver (Figure 4a), kidney (Figure 4b) weights and adiposity index change (Figure 4c) in normal and obese dyslipidemic rats. On day 120, the relative liver weight and adiposity index decreased slightly from 3.22 ± 0.19 to 2.92 ± 0.10% (LCD group) and from 0.14 ± 0.01 to 0.13 ± 0.01% (LCD + SADE group), and the difference was not statistically significant (*p* > 0.05). However, SADE administration in HCD animals significantly restored the relative liver weight (*p* < 0.05; Figure 4a) and adiposity index (*p* < 0.05; Figure 4c) in comparison to the HCD group. Though, the relative weight of the kidney decreased in HCD and HCD + SADE groups compared to LCD and LCD + SADE groups and showed a significant difference between HCD + SADE and LCD groups (*p* < 0.05; Figure 4b).

Saidi et al. [44] reported that HCD resulted in a significant increase in relative liver weight and adiposity index due to the accumulation of energy as triglycerides stored in tissues as well as its deposition in these organs. The increase in body weight of rats consuming high-energy diets is a sign of the increase in the number and/or size of adipocytes [57]. Adipose tissue is an active endocrine tissue secreting adipocytokines that affect full-body energy homeostasis through the sensing metabolic signals [57]. Many authors reported that the consumption of an HCD increases body weight and induces the accumulation of fat in adipose tissue. The accumulation of fat is the result of direct excess intake of a high-fat diet and/or the synthesis of fatty acids, mainly from carbohydrates [50]. The oral administration of SADE induced a decrease in body weight and adiposity index. A similar effect was shown by Chinchu et al. [5], who reported that 3.23 g/kg of *Varanadi kashayam* decocted extract for 6 weeks resulted in a significant decrease in organ weight compared to that of the high-fat diet group.

### 3.5. Effects of SADE on Blood Glucose and Serum Lipids Parameters

#### 3.5.1. Effects of SADE on Blood Glucose Level

Table 3 shows the average blood glucose level measured during 120 days in animal groups receiving LCD, LCD + SADE, HCD and HCD + SADE. At the baseline, the glycaemic level was similar between the different groups. From day 45 until the end of experimentation, glycemia values were significantly increased in the HCD group (140 ± 5 mg/dL) compared to the LCD (81 ± 4 mg/dL), LCD + SADE (65 ± 5 mg/dL) and HCD + SADE groups (73 ± 7 mg/dL) (*p* < 0.05) but animals remain not diabetic. SADE administration seems to modulate glycemia levels. Indeed, phenolic compounds possess redox potential and may act as antioxidants inducing hypoglycemic effects by enhancing glucose uptake [58].

#### 3.5.2. Effects of SADE on Serum Lipid Profile

**Figure 5:**
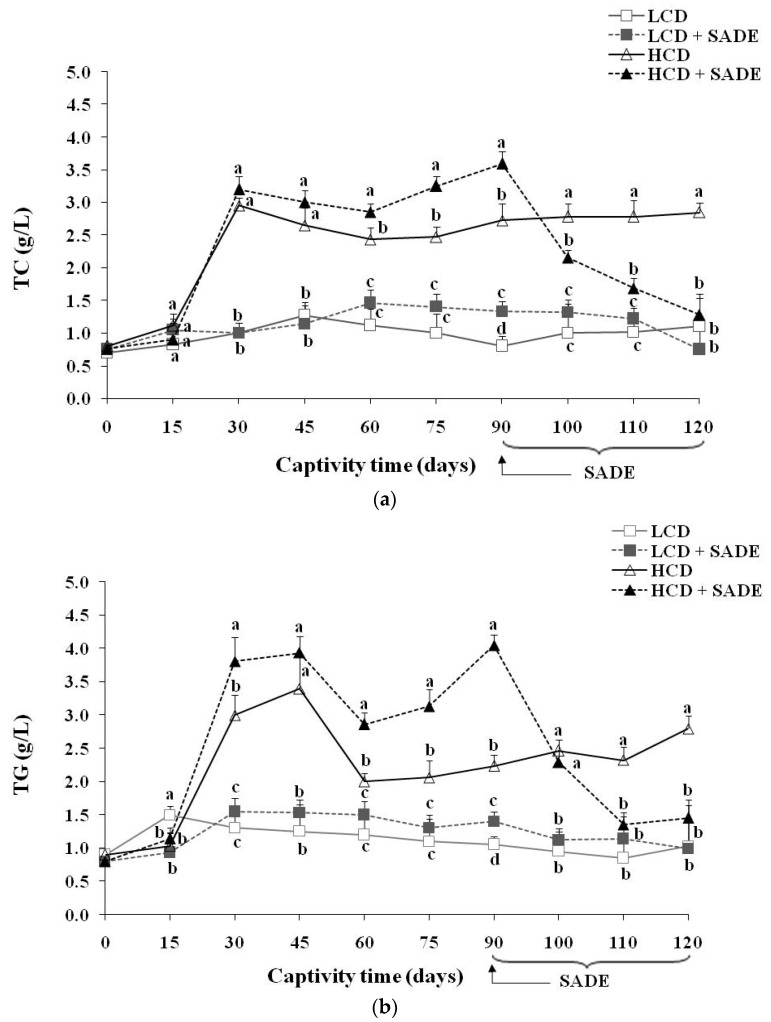

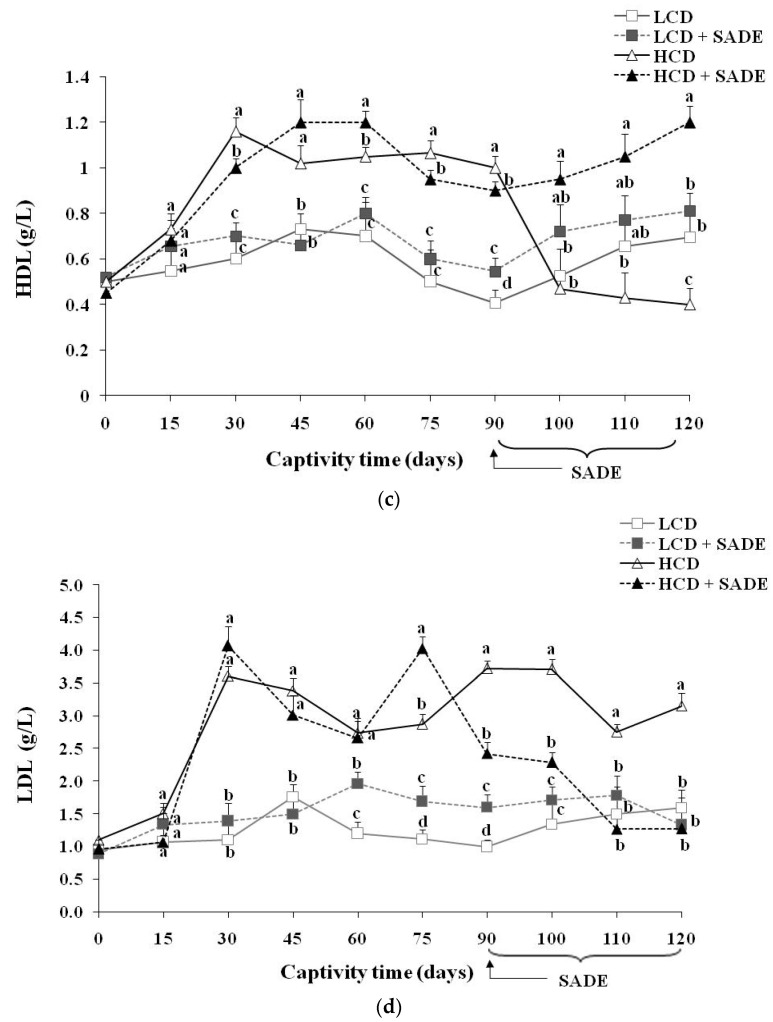

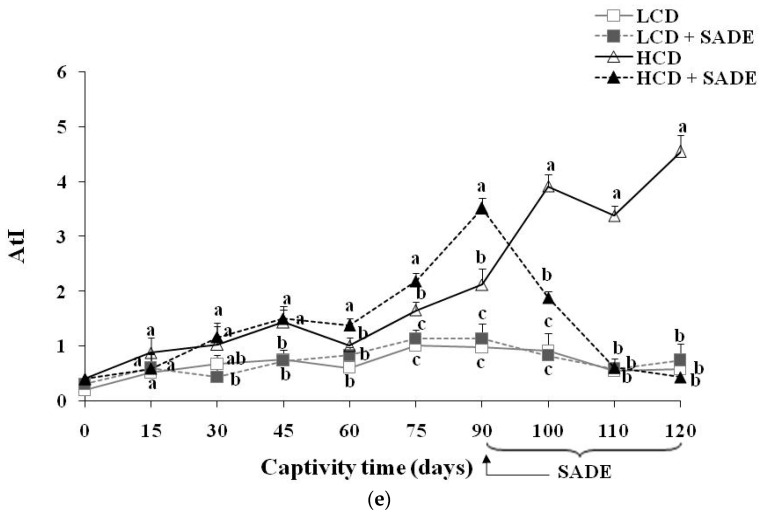
(**a**) Total cholesterol (TC) (g/L) in *P. obesus*. LCD: animals group fed with natural low-calorie vegetable diet, LCD + SADE: LCD treated with *S. arabica* decocted, HCD: rats fed with high-calorie diet and HCD + SADE: rats fed with high-calorie diet treated with *S. arabica* decocted extract. (**b**) Triglycerides (TG) (g/L) in *P. obesus*. LCD: animals group fed with natural low-calorie vegetable diet, LCD + SADE: control treated with *S. arabica* decocted, HCD: rats fed with high-calorie diet and HCD + SADE: rats fed with high-calorie diet treated with *S. arabica* decocted extract. (**c**) HDL (g/L) in *P. obesus*. LCD: animals group fed with natural low-calorie vegetable diet, LCD + SADE: LCD group treated with *S. Arabica* decocted, HCD: rats fed with high-calorie diet and HCD + SADE: rats fed with high-calorie diet treated with *S. arabica* decocted extract. (**d**) LDL (g/L) in *P. obesus*. LCD: animals group fed with natural low-calorie vegetable diet, LCD + SADE: LCD treated with *S. arabica* decocted, HCD: rats fed with high-calorie diet and HCD + SADE: rats fed with high-calorie diet treated with *S. arabica* decocted extract. (**e**) Atherogenic index (AtI) in *P. obesus*. LCD: animals group fed with natural low-calorie vegetable diet, LCD + SADE: LCD group treated with *S. arabica* decocted extract, HCD: rats fed with high-calorie diet and HCD + SADE: rats fed with high-calorie diet treated with *S. arabica* decocted extract. Results are presented as means ± SD for ten rats in each group. Means not sharing the same superscript letters (a, b, c, d) for each captivity time are significantly different between groups (Tukey’s post hoc test, *p <* 0.05). “a” denotes the highest value and “d” represents the lowest value.

The results of serum lipid contents are shown in Figure 5. The serum lipid profile of the LCD and LCD + SADE animals remained almost stable during the 120 days of the experiment. The *P. obesus* subjected to an HCD showed a highly significant increase after 120 days of treatment in TC (*p* < 0.05) (Figure 5a), TG (*p* < 0.05) (Figure 5b), LDL (*p* < 0.05) (Figure 5d), atherogenic index (AtI) (*p* < 0.05) (Figure 5e) and a decrease in the levels of HDL (*p* < 0.05) (Figure 5c) compared to LCD, LCD + SADE and HCD + SADE groups. A high level of HDL was observed in HCD and HCD + SADE groups during the first three months. This is consistent with several previous studies which have shown that HCD (3.25–3.70 kcal/g) administration in *P. obesus* induced the development of obesity after two to three months and metabolic syndrome such as dyslipidemia after 16 weeks. Indeed, the metabolic syndrome and dyslipidemia seem to induce insulin resistance in peripheral tissues leading to an enhanced hepatic flux of fatty acids and forming adipose tissue resistant to the anti-lipolytic effects of insulin. High levels of serum TG observed in HCD groups are generally associated with increased VLDL secretion by which lipolysis could produce HDL [11]. This may explain the high levels of HDL observed in HCD rats during the 90 days before the SADE administration. The authors also noticed a decrease in HDL levels ranging from 15–30% following the end of long-term exposure to the HCD [28,53]. The prominent decrease of HDL in the HCD group may be attributed to the disturbances in lipid and associated lipoprotein metabolism and the advanced dyslipidemia stage reached on day 90. Many authors reported a strong correlation between overweight or generalized obesity assessed using BMI and the decrease in HDL [59,60,61,62]. It is well known that the increase in blood biochemical parameters such as TC, LDL, and TG and the decrease of HDL after 90 days are dangerous indicators that develop the risk of cardiovascular complications such as dyslipidemia, atherosclerosis, coronary heart disease, and myocardial infarction [62,63].

The administration of SADE to HCD rats (HCD + SADE group) during 30 days (from day 90 to day 120) induced a significant decrease (*p <* 0.05) in serum lipid biochemical parameters (Figure 5) and in AtI value (Figure 5e) compared to those of obese and dyslipidemic rats (HCD group). AtI is a useful indicator of the risk of cardiovascular complications [5]. In this study, HCD + SADE significantly reduced the AtI compared to HCD (*p <* 0.05) (Figure 5e), and this indicates its cardio protective potential. At the end of treatment (on the 120th day), no significant difference was observed between the main biochemical lipid parameters of HCD + SADE group compared to the LCD and LCD + SADE groups.

Several approaches are proposed to reduce or suppress obesity and dyslipidemia, among them the use of natural herbal products with antioxidant activity [57]. *S. arabica* is not well investigated for its therapeutic use. Several species of *Salicornia*, such as *S. herbacea*, *S. bigelovii,* and *Sarcocornia perennis*, have been reported as presenting beneficial effects in vitro and in vivo, including antioxidant activity [25,43], hypolipidemic [23,64], anti-obesity [24,36], and immunomodulatory effects [65]. SADE resulted in rapid restoration of TC (Figure 5a), TG (Figure 5b), and LDL levels (Figure 5d). Indeed, a significant decrease from day 90 to day 120 in the level of TC, TG, and LDL was observed under the effect of SADE (HCD + SADE group). An increase in HDL (Figure 5c) was also observed in HCD + SADE group on day 120; it is well-known that a high serum level of HDL is a protective factor against vascular diseases. This result suggested that SADE exerts its anti-dyslipidemic effect (hypocholesterolemic and hypotriglyceridemic) on the HCD group. Similar studies reported the anti-hyperlipidemic effect of the *S. bigelovii* seed polysaccharide extract at 200 mg/kg body weight/day in hyper-cholesterol-fed rats [23]. Thus, in this study, it seems that SADE could have the capability to regulate lipid metabolism and the potential to reduce cardiovascular complications. Pichiah and Cha [24] reported a similar effect of *S. herbacea* supplementation in HCD rats. It has also been reported that administration of dried ethanolic extract of *S. herbacea* led to reducing weight gain and to a significant decrease of serum lipids in mice that exhibit type 2 diabetes and hyperlipidemia when prescribed for 10 weeks, with the suppression of genes linked to lipogenesis [64]. In addition, the flavonoids of this plant exert adipogenic inhibition in 3T3-L1 adipocytes [66]. Compared to other plants, oral administration of *Varanadi kashayam* decocted extract for a period of six weeks along with HCD to rats decreased the serum lipid profile [5]. Similar doses of *Ephedra alata* areal part (100 to 300 mg/kg/day) were reported to have positive effects on the reduction in blood lipid levels [67]. According to the literature, the hypocholesterolemic of SADE may be attributed to the catabolism of LDL and modulation of expression levels of genes related to lipid metabolism, as reported for other phenolic extracts [68]. Further molecular investigations will be completed to better understand the potent SADE role in the mechanism of regulation of lipids and its anti-obesity and anti-dyslipidemic effects. Recently, it was proved that phenolic compounds and phenolic extracts might contribute to reducing obesity and dyslipidemia by exerting different mechanisms. The main reported pathways of anti-obesity and dyslipidemic effects involving phenols are (i) enhancement of the in vivo antioxidant defense allowing protection of lipoprotein against oxidation and minimizing hepatic injury [13,14], (ii) inhibition of the key enzymes involved in carbohydrate (such as α-amylase) and fat metabolism (such as pancreatic lipase) which hamper the digestion and absorption of carbohydrates and fats in the small intestine [10,11], and (iii) decreasing of lipogenic adipogenic activities in liver and adipose tissues [1,9,44,69]. It was reported that gingerol [11] and *betula utilis* bark extract [10] induce the reduction of the absorption of fat and cholesterol by inhibiting the activity of pancreatic lipase. Similar effects were reported for gallic acid supplementation in rats fed with HFD. The improvement of antioxidant status contributes to reducing obesity. Similar effects were reported for the mixture extracts of *Morus alba* and *Aronia melanocarpa* against high-fat diet-induced obesity in C57BL/6J mice [9]. The authors showed that extract mixture exerts a synergistic effect against diet-induced obesity by decreasing expression levels of genes involved in lipid anabolism (SREBP-1c, PPAR-, CEBP, FAS, and CD36), increasing the expression levels of lipolysis-related genes in liver and adipose tissue and upregulated AMPK signaling. Feng et al. [1] showed, using transcriptome analysis and real-time quantitative RT-PCR, that heptamethoxyflavone supplementation in rats fed a high-fat diet markedly downregulated hepatic genes related to adipogenesis transcription and inflammatory responses and significantly upregulated genes related to fatty acid oxidation and energy expenditure. Similar hypolipidemic effects were reported for *Coriandrum sativum* L. in *Meriones shawi* rats fed high-fat diets [12].

### 3.6. Effects of SADE on Liver Enzyme Markers and Liver Histology

**Figure 6:**
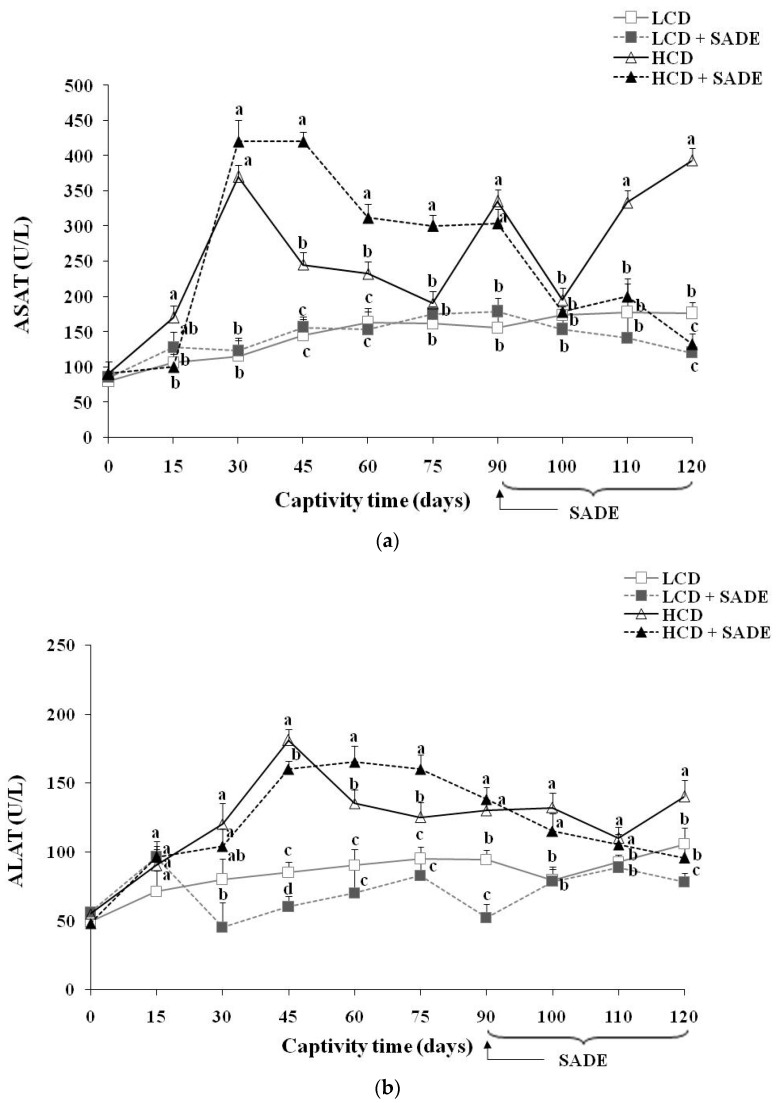
(**a**) ASAT activity (U/L) in *P. obesus*. LCD: animals group fed with natural low-calorie vegetable diet, LCD + SADE: LCD treated with *S. arabica* decocted, HCD: rats fed with high-calorie diet and HCD + SADE: rats fed with high-calorie diet treated with *S. arabica* decocted extract. Results are presented as means ± SD for ten rats in each group. Means not sharing the same superscript letters (a, b, c) for each captivity time are significantly different between groups (Tukey’s post hoc test, *p <* 0.05). “a” denotes the highest value and “c” represents the lowest value. (**b**) ALAT activity (U/L) in *P. obesus*. LCD: animals group fed with natural low-calorie vegetable diet, LCD + SADE: control treated with *S. arabica* decocted, HCD: rats fed with high-calorie diet and HCD + SADE: rats fed with high-calorie diet treated with *S. arabica* decocted extract. Results are presented as means ± SD for ten rats in each group. Means not sharing the same superscript letters (a, b, c, d) for each captivity time are significantly different between groups (Tukey’s post hoc test, *p <* 0.05). “a” denotes the highest value, and “d” represents the lowest value.

**Figure 7:**
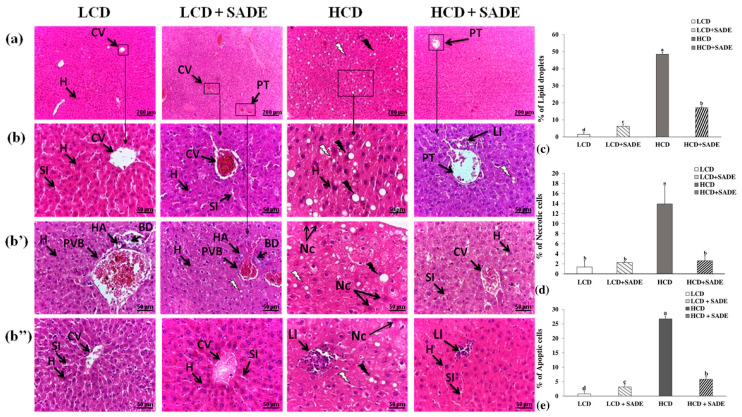
Histological analysis of liver tissues by hematoxylin-eosin (H&E) staining ((**a**): magnification, 10×, scale bar = 200 µm), ((**b**–**b’’**): magnification, 40×; scale bar = 50 µm). (**b**–**b’’**) represent the different regions of the same slide of each group. Liver sections of LCD: animals group fed with natural low-calorie vegetable diet and LCD + SADE: LCD group treated with *S. arabica* decocted showed the normal structure of hepatocytes with normal sinusoids and central vein and a normal portal with normal bile ducts (**a**–**b’’**). Liver sections of HCD: rats fed with a high-calorie diet demonstrated moderate alterations such as microvesicular and macrovesicular fatty degeneration of hepatocytes (**a**–**b’’**), inflammatory (**b’’**) and necrosis cells (**b’**,**b’’**) and liver sections of HCD + SADE: rats fed with high-calorie diet treated with *S. arabica* decocted extract reveal a regular aspect of hepatocytes near the normal liver and exhibiting attenuation of inflammation and steatosis (**a**–**b’’**). CV: centrilobular Vein, SI: sinusoids, H: hepatocyte, Nc: necrotic cell, PVB: portal vein branch, PT: Portal triad, HA: hepatic artery, BD: bile duct, LI: inflammatory leukocyte infiltrations. Black arrows indicate macrovesicular droplets and white arrows indicate microvesicular droplets. (**c**) Lipid droplet quantification expressed in percentage of the number of hepatic cells and are means ± SD for triplicate analyses. (**d**) Necrotic cells quantification expressed in percentage of the number of hepatic cells and are means ± SD for triplicate analyses. (**e**) Apoptic cells quantification expressed in the percentage of the number of hepatic cells and are means ± SD for triplicate analyses.

The serum levels of ASAT and ALAT corresponding to the four animal groups (LCD, LCD + SADE, HCD, HCD + SADE) are presented in Figure 6a,b. The histological changes of *P. obesus* liver tissues of animal groups assessed at the end of the experiment (four months) are shown in Figure 7. Compared to the LCD group, the activities of hepatic marker enzymes of ASAT (Figure 6a) and ALAT (Figure 6b) were significantly increased in the HCD group (*p* < 0.05). However, oral administration of SADE to obese and dyslipidemic *P. obesus* (HCD + SADE) induced a significant reduction in serum ALAT and ASAT on day 120 and showed no significant difference comparable to the LCD + SADE (Figure 6a) and LCD groups (Figure 6b).

The HCD-induced body weight increase (Figure 2) causes changes in lipid balance (Figure 5a–e) and promotes transaminase enzyme activities of ASAT (Figure 6a) and ALAT (Figure 6b), inducing impaired liver function compared to control rats. Several reports demonstrated that the increase in ASAT and ALAT is a principal indicator of liver dysfunction and disturbances in the biosynthesis of these enzymes, with an alteration in the permeability of the hepatic membrane [70]. Indeed, Spolding et al. [71] showed that the *P. obesus* fed a cholesterol-supplemented standard rodent diet for four weeks, causing a significant increase in ASAT and ALAT levels. Antioxidants of SADE seem to reduce serum ASAT and ALAT, which represents a clear indication of the improvement of the functional status of the liver. Therefore, treatment with 300 mg SADE/kg per day for a month moderated the deleterious effects of the HCD. Indeed, SADE induced a significant decrease in the activities of ASAT (Figure 6a), and ALAT (Figure 6b) compared to the HCD group. These results are in agreement with those of Gargouri et al. [65]. The authors proved that treated rats with a dried extract of *Sarcocornia perennis* regulates the enzyme levels (ASAT and ALAT) and reduces the cell oxidative damage induced by lead. It is also reported that an aqueous extract of *Salicornia* shows an hepato-protective effect at a dose of 500 mg/kg in mice stressed by acetaminophen [72].

The increase of ASAT and ALAT activity levels and their decrease after SADE supplementation indicate the restoring effect of SADE supplementation, and this is in agreement with the analysis of the histology of the liver tissue (Figure 7). Indeed, the liver tissues of LCD and LCD + SADE groups presented normal architecture, with radiating organization of hepatocytes from the central vein and showed a normal portal triad (Figure 7a–b’’). Hepatic injury was observed in the liver tissue of the animals of HCD groups (Figure 7a–b’’). It was marked by strong ballooning hepatocytes characterized by enlarged size, pale color and the presence of numerous micro and macro-vesicular and intracellular lipid droplets. In addition, the liver sections of HCD groups (Figure 7b–b’’) showed very severe hepatotoxicity with hepatocyte necrosis and leukocyte inflammatory infiltration, mainly in the lobular and portal levels. The percentage of apoptotic cells, necrotic cells and lipid droplets (Figure 7c–e) was significantly increased in the liver tissue of the HCD group. The administration of SADE (HCD + SADE group) seems to be able to partially restore the hepatic morphology back to a normal state (regular size of hepatocytes, attenuation of inflammation and steatosis) and significantly decrease the percentage of apoptotic cells, necrotic cells and lipid droplets. Besides, oral administration of SADE didn’t induce any hepatotoxicity signs and limited HCD-induced hepatic steatosis. Similar observations were reported in HCD-induced obese *P. obesus* [44] after supplementation of spirulina. Similarly, Hsu et al. [13] reported the attenuation of inflammation and steatosis after antioxidant supplementation and this positive effect was attributed to the amelioration of oxidative status in the liver tissue.

### 3.7. Principal Components Analysis of Biochemical Parameters, Body Weight Gain

**Figure 8:**
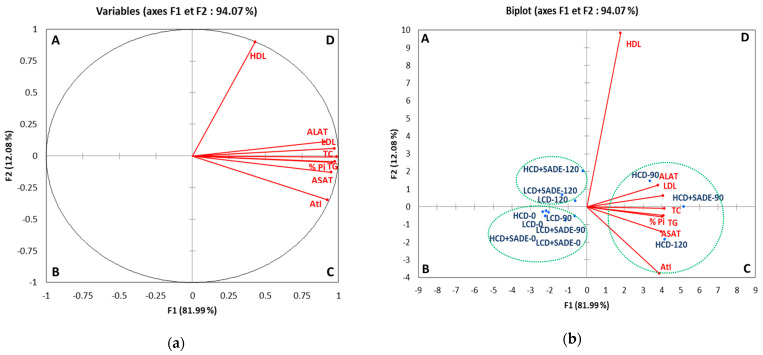
Biplot of objects and component loads for grouping of biochemical descriptors (TC, TG, HDL, LDL, AtI, ASAT, ALAT, and % Pi) (**a**) and animal groups samples (**b**). LCD: animals group fed with natural low-calorie vegetable diet, LCD + SADE: LCD treated with *S. arabica* decocted, HCD: rats fed with high-calorie diet and HCD + SADE: rats fed with high-calorie diet treated with *S. arabica* decocted extract. TC: Total cholesterol, TG: Total triglycerides, LDL: Low-density lipoprotein, HDL: High-density lipoprotein, AtI: Atherogenic index, ASAT: Aspartate aminotransferase, ALAT: Alanine aminotransferase.

Figure 8 showed the PCA biplot performed on body weight gain (% Pi), biochemical parameters (TG, TC, HDL, LDL, AtI) and ASAT and ALAT activities assessed for different animal groups on day 0 (the beginning of the experiment), day 90 (the beginning of SADE administration) and day 120 (the end of SADE administration and of the experiment). The biplot revealed that PCA described ~94% of the whole data variation through the first two components; respectively, PC1 explained 82% of the variance, and PC2 accounted for an additional 12% of the variance (Figure 8a,b). The first dimension was represented positively by % Pi (0.976), AtI (0925), LDL (0.972), TG (0.958), TC (0.988), ASAT (0.950) and ALAT (0.907). The biplot was divided into four quadrants (A, B, C, and D), where three clusters comprising animal groups with similar biochemical parameters, % Pi and the atherogenic index, and exhibiting similar ranges of ASAT and ALAT activities are distinguished. The first cluster (quadrant A) comprises LCD, LCD + SADE and HCD + SADE animal groups on day 120. The second group (quadrant B) contains the four animal groups (LCD, LCD + SADE, HCD, HCD + SADE) on day 0 (the beginning of the experiment) and two LCD groups on day 90 (LCD and LCD + SADE). The third group (quadrants C and D) is represented by three animal groups (HCD and HCD + SADE on day 90) and HCD on day 120. The latter cluster is positively correlated to % Pi, ASAT and ALAT activities and lipid parameters. Table 4 shows the correlation matrix between all measured parameters. The average body weight gain, % Pi, is positively correlated to AtI, all lipid biochemical parameters and ASAT and ALAT (R^2^ ≥ 0.836). At the same time, a weak correlation was recorded with HDL content (0.383 ≤ R^2^ ≤ 0.476). Strong positive correlations (R^2^ ≥ 0.809) were also observed between AtI, all lipid parameters (except HDL) and ALAT and ASAT. These results are in agreement with biochemical analyses shown above and confirm the strong positive correlation between TC, TG, HDL, LDL, % Pi and levels of hepatic enzymes markers, whereas HDL seems to be weakly correlated to all measured parameters.

## 4. Conclusions

One-month oral administration of 300 mg of the *S. arabica* decocted extract (SADE)/kg per day to obese and dyslipidemic *P. obesus* fed a high-calorie diet induced a significant decrease in body weight, body mass index, food intake and energy intake, liver relative weight and adiposity index. SADE supplementation in the *P. obesus* diet induces, in the high-calorie diet animals group, significant hypocholesterolemic and hypotriglyceridemic effects with a significant decrease in atherogenic index. It decreases aspartate aminotransferase and alanine aminotransferase levels and significantly reduces liver tissue damage. SADE acts positively to modulate lipid metabolism disturbance and liver injury in *P. obesus*. The results suggest that SADE has the potential to be a suitable candidate for further investigations as an anti-obesity and hypolipidemic natural agent. The molecular and cellular mechanisms (i.e., involvement in the regulation of gene expression related to lipid metabolism and enhancement of liver antioxidant status) and the active phenolic compounds responsible for these activities remain to be elucidated.

## Figures and Tables

**Table 1:** Proximate chemical composition of LCD and of HCD.

	Diets	
Components	LCD	HCD
Moisture	81.63 ± 0.69 ^a^	9.11 ± 0.38 ^b^
Fat	0.48 ± 0.08 ^b^	18.91 ± 0.08 ^a^
Ash	8.42 ± 0.15 ^a^	2.83 ± 0.23 ^b^
Proteins	3.09 ± 0.22 ^b^	10.82 ± 0.82 ^a^
Carbohydrates	6.38 ± 0.41 ^b^	58.33 ± 1.32 ^a^
Energetic value ^§^	0.42 ± 0.03 ^b^	4.50 ± 0.02 ^a^

g/100 g wet basis. §: kcal/g wet basis. LCD: Low-Calorie Diet, and HCD: High-Calorie Diet. Results are present as means ± S.D for triplicate analysis. Values with different letters are statistically different (*p <* 0.05).

**Table 2:** Phenols, flavonoids and antioxidant activity of *Salicornia arabica* decocted extract (SADE).

Parameters	TPC	TFC	DPPH	ABTS
SADE	20.50 ± 0.30	18.20 ± 0.20	3.20 ± 0.10	17.31 ± 0.65

TPC (mg GAE/g dried extract): Total phenols content, GAE: Gallic Acid Equivalent, TFC (mg QE/g SADE): Total flavonoids content, QE: Quercetin Equivalent, DPPH-Radical scavenging activity and ABTS-radical scavenging assay (mg Trolox Equivalent/g). SADE: *S. arabica* decocted extract. Results are present as means ± S.D. for triplicate analysis.

**Table 3:** Effect of SADE on glycemia (mg/dL) variation in *P. obesus*.

Day	Glycaemia (mg/dL)			
	LCD	LCD + SADE	HCD	HCD + SADE
0	72 ± 10 ^aAB^	67 ± 15 ^aA^	82 ± 3 ^aB^	86 ± 5 ^aAB^
15	76 ± 5 ^aAB^	68 ± 7 ^aA^	89 ± 17 ^aB^	82 ± 14 ^aAB^
30	73 ± 15 ^aAB^	58 ± 11 ^aA^	75 ± 5 ^aB^	76 ± 9 ^aB^
45	78 ± 2 ^bcAB^	63 ± 4 ^cA^	93 ± 7 ^abB^	99 ± 7 ^aA^
60	90 ± 8 ^aA^	70 ± 10 ^aA^	93 ± 13 ^aB^	85 ± 8 ^aAB^
75	73 ± 7 ^bAB^	66 ± 6 ^bA^	96 ± 7 ^aB^	81 ± 10 ^aA^
90	73 ± 8 ^aAB^	74 ± 10 ^aA^	89 ± 17 ^aB^	96 ± 8 ^aA^
100	61 ± 1 ^cB^	62 ± 2 ^cA^	96 ± 1 ^aB^	72 ± 2 ^bB^
110	73 ± 5 ^bAB^	60 ± 5 ^bA^	109 ± 23 ^aAB^	70 ± 2 ^bB^
120	81 ± 4 ^bAB^	65 ± 5 ^cA^	140 ± 5 ^aA^	73 ± 7 ^bcB^

LCD: animals group fed with natural low-calorie vegetable diet, LCD + SADE: LCD treated with *S. arabica* decocted, HCD: rats fed with high-calorie diet and HCD + SADE: rats fed with high-calorie diet treated with *S. arabica* decocted extract. Results are presented as means ± SD for ten rats in each group. Means not sharing the same superscript letters (a, b, c) in a row are significantly different between groups (Tukey’s post hoc test, *p* < 0.05). Means not sharing the same superscript letters (A, B, C) in a column is significantly different between groups (Tukey’s post hoc test, *p* < 0.05). “A, a” denotes the highest value and “C, c” represents the lowest value.

**Table 4:** PCA Pearson correlation matrix of measured parameters: % Pi, biochemical parameters (TG, TC, HDL, LDL, AtI), ASAT and ALAT levels.

Variables	% Pi	AtI	LDL	HDL	TG	TC	ASAT	ALAT
% Pi	1	0.913	0.928	0.383	0.978	0.980	0.897	0.836
AtI	-	1	0.864	0.096	0.892	0.904	0.934	0.809
LDL	-	-	1	0.466	0.927	0.979	0.921	0.847
HDL	-	-	-	1	0.364	0.417	0.297	0.476
TG	-	-	-	-	1	0.974	0.841	0.806
TC	-	-	-	-	-	1	0.916	0.844
ASAT	-	-	-	-	-	-	1	0.888
ALAT	-	-	-	-	-	-	-	1

TC: Total cholesterol, TG: Total triglycerides, LDL: Low-density lipoprotein, HDL: High density lipoprotein, AtI: Atherogenic index, ASAT: Aspartate aminotransferase, ALAT: Alanine aminotransferase.

## Data Availability

The data that support the findings of this study are available from the corresponding author upon reasonable request.
